# The Role of Physical Stimuli on Calcium Channels in Chondrogenic Differentiation of Mesenchymal Stem Cells

**DOI:** 10.3390/ijms19102998

**Published:** 2018-10-01

**Authors:** Ilona Uzieliene, Paulius Bernotas, Ali Mobasheri, Eiva Bernotiene

**Affiliations:** 1 Department of Regenerative Medicine, State Research Institute Centre for Innovative Medicine, LT-08406 Vilnius, Lithuania; ilona.uzieliene@imcentras.lt (I.U.); bernotaspaul@gmail.com (P.B.); a.mobasheri@surrey.ac.uk (A.M.); 2 Department of Veterinary Pre-Clinical Sciences, School of Veterinary Medicine, Faculty of Health and Medical Sciences, University of Surrey, Guildford GU2 7AL, UK; 3 Arthritis Research UK Centre for Sport, Exercise and Osteoarthritis, Queen’s Medical Centre, Nottingham NG7 2UH, UK

**Keywords:** human mesenchymal stem cells, calcium channels, chondrogenic differentiation, electrical stimulation, electromagnetic field, magnetic field, mechanical stimulation

## Abstract

Human mesenchymal stem cells (hMSC) are becoming increasingly popular in tissue engineering. They are the most frequently used stem cell source for clinical applications due to their high potential to differentiate into several lineages. Cartilage is known for its low capacity for self-maintenance and currently there are no efficient methods to improve cartilage repair. Chondrogenic differentiation of hMSC isolated from different tissues is widely employed due to a high clinical demand for the improvement of cartilage regeneration. Calcium channels that are regulated by physical stimuli seem to play a pivotal role in chondrogenic differentiation of MSCs. These channels increase intracellular calcium concentration, which leads to the initiation of the relevant cellular processes that are required for differentiation. This review will focus on the impact of different physical stimuli, including electrical, electromagnetic/magnetic and mechanical on various calcium channels and calcium signaling mechanisms during chondrogenic differentiation of hMSC.

## 1. Introduction

The poor regenerative capacity of human articular cartilage is a major challenge for researchers and clinicians that are working in the area of cartilage repair and it has gained particular attention during the past decades. The degradation of cartilage tissue, caused by trauma or chronic and progressive degenerative joint disease, e.g., osteoarthritis (OA) or rheumatoid arthritis (RA), has become a global problem for which no efficient therapy is available [1]. The ultimate goal of all cartilage regeneration techniques is the reconstruction of mature, structurally organized, and biomechanically compliant hyaline cartilage. Although the majority of cartilage repair techniques produce poor outcomes with limited success rates, the cell-based therapies, such as application of multipotent mesenchymal stem cells (MSCs), seem promising candidates for cartilage regeneration [2]. Human MSCs are adult stem cells which are characterized by self-renewal, high proliferation capability, production of clonal cell populations, and multilineage differentiation. Due to their significant role in tissue repair and regeneration MSCs became among the most valuable objects in regenerative medicine. MSCs can be isolated from different sources, including bone marrow, adipose tissue, synovial membrane, umbilical cord, dental pulp, menstrual blood, and others. They are plastic- adherent cells characterized by expression of typical surface markers CD73, CD90, CD105 and lack of expression of hematogenic surface markers CD14, CD34, and CD45 and they have a potential to differentiate at least into chondrogenic, osteogenic and adipogenic lineages when cells are cultured under defined conditions in vitro. The potential of MSCs to differentiate into chondrogenic lineage make them very useful tools for understanding the regulation of chondrogenesis process and maintenance of cartilage tissue integrity, furthermore, MSCs seem attractive candidates for cartilage tissue engineering for therapeutic applications [3].

Differentiation of MSCs includes multiple stages that generate specific proteins according to each lineage. A number of biological/chemical factors can regulate the differentiation process. For instance, steroid hormones and growth factors, such as tumor growth factors-β (TGF-βs), Activin A, and bone morphogenetic proteins (BMP)-2,-4,-7 are crucial to induce chondrogenesis and synthesize collagen type 2, aggrecan and other extracellular matrix (ECM) proteins [4], In many of these processes, calcium ions (Ca^2+^) play a crucial role in regulating cell functions [5], whereas the increase of intracellular Ca^2+^ was shown to improve cell differentiation potential [6].

Although Ca^2+^ can enter cells through different calcium channels, primarily voltage operated calcium channels (VOCCs), transient receptor potential (TRP) channels, and purinergic receptors have been shown to be regulated by physical stimuli [5,6,7,8]. Several studies demonstrated that the inhibition of VOCCs in stem cells leads to a poor chondrogenesis, especially on its early stages. This highlights an important role for VOCCs in cartilage development. Moreover, the application of VOCCs inhibitors, which are widely used as therapeutic drugs in cardiovascular diseases (e.g., verapamil, nifedipine, amlodipine, nicardipine) may also result in attenuated cartilage degradation and the prevention of OA progression [9]. Members of the TRP channel superfamily were also shown to be involved in chondrogenesis induction through activation of the SOX9 pathway [10]. Therefore, stimulation of these calcium channels opens a new research area, which may lead to the development of innovative approaches to improve chondrogenic differentiation in vitro, and, most importantly, cartilage repair in vivo. This review presents an up-to-date overview of the role of physical stimuli on calcium channels in chondrogenic differentiation of MSCs. We have focused on three different types of physical stimuli that have been described to influence calcium channel activation—electric, electromagnetic/magnetic, and mechanical forces.

## 2. Calcium Signaling in Cells

Intracellular Ca^2+^ levels control a number of crucial processes in cells, including exocytosis, apoptosis, motility, gene transcription, and differentiation [11]. Intracellular Ca^2+^ can diffuse into mitochondrias or nucleus, where interplays in several counteracting processes, which are then divided into Ca^2+^ “on” and “off” processes, depending whether they increase or decrease intracellular Ca^2+^. When the cells are in their “resting” state, Ca^2+^ concentration balance lies in favor to “off” processes, decreasing Ca^2+^ up to 100 nmol/L [12]. However, when the cells are being stimulated by different factors (e.g., mechanical forces, electric stimulation, magnetic field), “on” processes are being activated and intracellular Ca^2+^ can be increased up to 1 µmol/L. Intracellular Ca^2+^ binds to different proteins that take part in Ca^2+^ signaling pathways [12]. One of these proteins are calmodulin, which is involved not only in Ca^2+^ metabolic pathway induction, but also in silencing Ca^2+^ signaling, 4, 5 bisphosphate (PIP2), C2 domain, calcineurin, and others [13]. Most of the cells utilize two main Ca^2+^ sources for generating intracellular signals, one of which is Ca^2+^ entry through the plasma membrane and the other is the release of intracellular Ca^2+^ stores. Intracellularly Ca^2+^ is stored in the endoplasmic reticulum, where two main channels, namely, inositol 1,4,5-trisphosphate receptor (InsP3R) and ryanodine receptor (RyR), may release Ca^2+^ if its concentration is getting low [14]. Ca^2+^ transport across the plasma membrane occurs via two distinct pathways—VOCCs, and agonist-dependent Ca^2+^ entry pathways, which include a number of different Ca^2+^ channels.

## 3. VOCCs and Their Regulation

**Figure 1:**
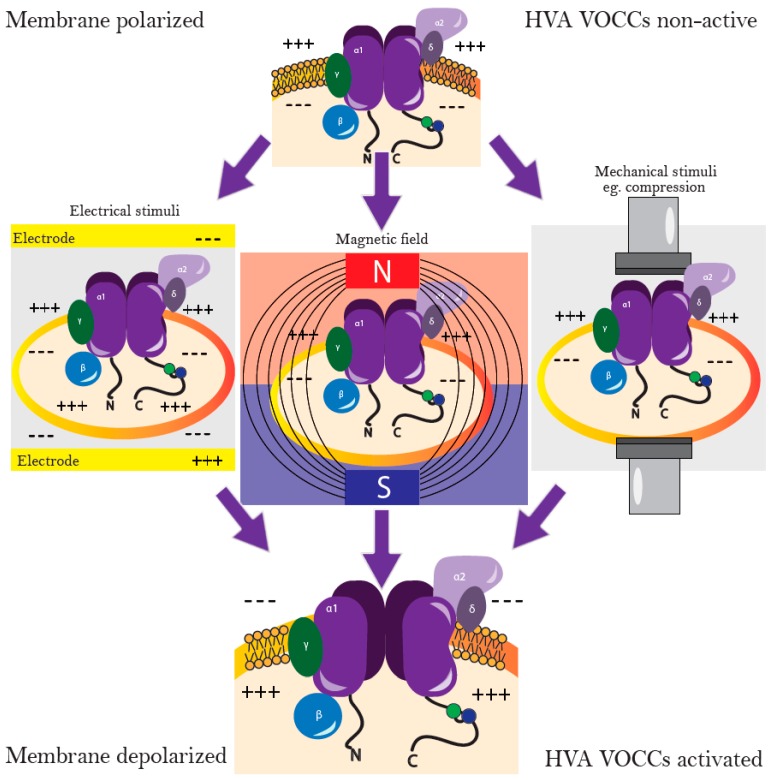
Different stimuli impact on high-voltage activated (HVA) voltage-operated calcium channels (VOCCs). N-N-terminus of the protein (NH2), C-C-terminus of the protein (COOH).

**Figure 2:**
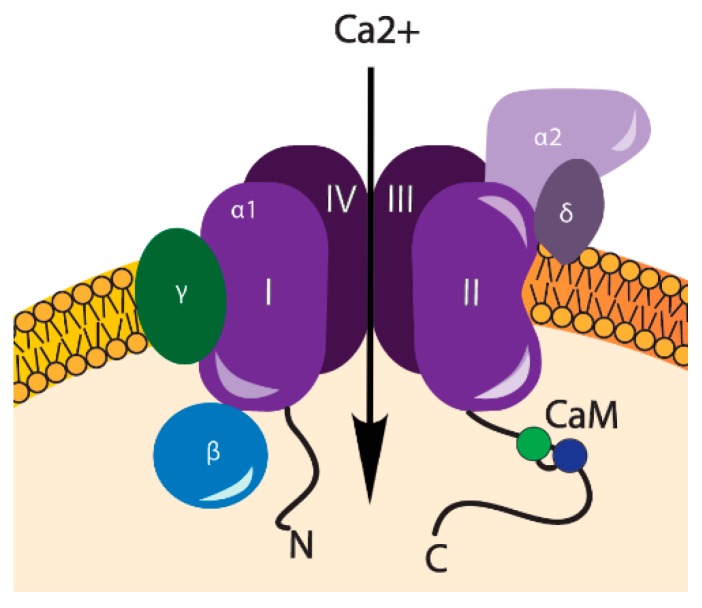
Structure of high-voltage activated calcium channel. α1—a central pore-forming subunit, α2δ subunit—a disulfide-linked glycoprotein dimer, an intracellular β subunit, a transmembrane glycoprotein γ subunit, and a calmodulin (CaM) bound to the C-terminal cytoplasmic tail [19].

VOCCs comprise a group of membrane proteins that are predominantly found in “excitable” cells (cardiomyocytes, muscle, neurons, glial cells). These channels are well-known for their involvement in electrical current generation, however, they are also abundantly expressed in a range of “non-excitable” cells, including osteoblasts and chondrocytes [5]. VOCCs are highly selective for Ca^2+^, even though Na^+^ concentration in an extracellular space is 70-fold higher [15]. VOCCs are activated by membrane depolarization and they are assigned into two groups, according to the membrane potentials, at which they are opened, including low-voltage activated (LVA) and high-voltage activated (HVA) [16]. The LVA channels, also often called as T-type channels, are opened in response to small physical changes from the resting membrane potential, whereas HVA are activated by stronger depolarizations, caused by mechanical, electrical, or chemical stimuli [17], see Figure 1. HVA channels are subdivided into L-, N-, P/Q-, and R-type channels and have been characterized on the basis of a number of criteria, including single channel conductance, kinetics, pharmacology, and cellular distribution [16]. Due to their exclusive importance in intracellular Ca^2+^ homeostasis, L-type channels are considered to be the major HVA channels. They are the main route for calcium influx in cardiac, skeletal, and smooth muscle cells [16]. All HVA VOCCs are tetrameric proteins, consisting of four transmembrane domains with four subunits (α1, α2δ, β and γ). The α1 subunit forms a pore, which determines the type of the channel. α2δ, β, and γ subunits modulate the functional properties of VOCCs. Calmodulin (CaM) is a ubiquitous calcium binding protein bound to the I-II loop of the CaVs and is also a part of VOCCs, involved in a further Ca^2+^ signaling [18] (Figure 2).

α1 subunits may be different among VOCCs, depending on the difference of Ca^2+^ currents. These subunits are divided into three functionally and structurally related proteins—CaV1, CaV2, and CaV3 [19] (Table 1).

Several studies have shown that other types of channels that are closely involved in mechanotransduction are TRPs [8,17]. They are widely expressed in a large number of different tissues, and, when activated, they cause cell depolarization, which may in turn trigger a plethora of VOCCs. By changing the membrane potential and the local Ca^2+^ gradients, TRP channels contribute to the modulation of the driving force for Ca^2+^ entry and provide intracellular pathways for Ca^2+^ release from cellular stores. They obviously play an important role in the regulation of the activity for voltage-dependent ion channels, including VOCCs [20,21,22].

## 4. Transient Receptor Potential Channels

TRP channels are ion channels that are induced by various physical and chemical stimuli, ranging from PIP2, Ca^2+^ and cyclic nucleotides to temperature, osmotic pressure, and other various environmental inputs [23]. When activated, TRP channels allow for the entry of Ca^2+^ into the cell, in turn increasing its intracellular concentration and depolarizing the cell. TRP channels are formed by six transmembrane domains (TMs) and categorized into seven subfamilies: TRPA, TRPC, TRPM, TRPML, TRPN, TRPP, and TRPV. As TRP channels are prevalent in almost all mammalian cells and are involved in regulation of Ca^2+^ concentration and signaling is significant in both excitable and non-excitable cells [24]. Some TRP channels found in the plasma membrane of MSCs were shown to play a pivotal role in chondrogenesis. For instance, TRP vanilloid 4 (TRPV4), activates SOX9 pathway by increasing the levels of SOX9 mRNA and protein, as well as essential cartilage-specific extracellular matrix molecules type II collagen and aggrecan [25]. Other channels that were found in MSCs include some types of channels that belong to the TRPC subfamily, with the noticeable significance of TRPC1, which contributes to the proliferation of these cells and early chondrocyte expansion [26]. Blocking both TRPV4 and TRPC1 channels by their inhibitors (2-aminoethoxydiphenylborane (2-APB) and Ruthenium Red, respectively) at the initial stages of chondrogenic differentiation of BMMSCs effectively blocked the chondrogenesis entirely [10].

## 5. Purinergic Receptors (P2X4)

Purinergic receptors are activated by purines or their nucleotides. Their function is mostly associated with purinergic signaling pathways that are involved in neurological processes [27], however, a purinergic receptor family P2X are ligand-gated ion channel receptors, which can cause Ca^2+^ influx into the cell in response to interactions with ATP molecules. P2X4 in particular was shown to regulate Ca^2+^ entry into the cytosol in chicken mesenchymal cells, which was linked to cartilage formation [28] and contribute to chondrogenic processes by increasing the Ca^2+^ concentration that is needed for hMSC differentiation [9]. The P2X receptor family and P2X4 in particular is associated with mechanotransduction and are able to detect mechanical forces, however, it is not known whether or not it is a mechanosensitive channel and how it contributes to the process of mechanical signaling [29].

## 6. Calcium Oscillations during Chondrogenic Differentiation of MSCs

The role of Ca^2+^ signaling in MSCs has gained increasing interest during the past decades. In 2002, Kawano and colleagues were the first to describe Ca^2+^ signaling in MSCs, suggesting that Ca^2+^ plays an important role in controlling many cellular processes, including cell growth, fertilization, transformation, secretion, smooth muscle contraction, sensory perception, and neuronal signaling [13]. Particular attention has been paid to the pathways that lead to those intracellular Ca^2+^ oscillations in MSCs and the transporters that may be responsible for those oscillations, as reviewed elsewhere [13]. However, some controversial data exist. Spontaneous Ca^2+^ oscillations in undifferentiated hMSCs in the absence of any agonist stimulations have been observed, suggesting that Ca^2+^ oscillations are predominantly referred to Ca^2+^ release from intracellular stores, but not to the Ca^2+^ entry through plasma membrane [5]. During osteogenic differentiation, no differences were observed between human MSCs that were treated with and without nifedipine, raising doubts about the involvement of L-type Ca^2+^ channels in osteogenesis [30]. However, nifedipine administered at 6 mg/kg daily was shown to reduce osteogenesis in a study on rabbits [31]. Furthermore, it significantly suppressed osteogenic differentiation of human periodontal ligament stem/progenitor cell line in vitro, treated with 5 mM CaCl_2_ [32], which suggests that extracellular Ca^2+^ entry through VOCCs might modulate the differentiation capacity of different cell types.

The crucial role of intracellular ion homeostasis in chondrocyte function has been extensively demonstrated [33]. Mature chondrocytes deploy a whole range of ion channels, including voltage-dependent potassium channels (KV), ATP dependent potassium channels (KATP), large and small conductance calcium-activated potassium channels (BK and SK), transient receptor potential channels (primarily TRPV1 and TRPV4), purinergic receptors (both P2X and P2Y subfamily members), voltage-gated and epithelial sodium channels, chloride channels, and also VOCCs [9]. This collection of ion channels in chondrocytes is required for the efficient extracellular matrix (ECM) turnover and homeostasis. Chelating free Ca^2+^, inhibiting voltage-gated calcium channels, and depleting intracellular calcium stores suppressed the beneficial effect of hydrostatic pressure on chondrogenesis, indicating that Ca^2+^ mobility might play an important role in the mechanotransduction of mechanical pressure [33,34]. During chondrogenic differentiation of MSCs, intracellular Ca^2+^ is known to be involved in wide range of Ca^2+^ sensitive signaling pathways, including various protein kinase systems, which may enable distinct gene expression profiles via differential activation of key transcription factors (NFAT, CREB, etc.), giving rise to chondrogenic differentiation [35]. Among all protein kinases, protein kinase C (PKC) play pivotal roles in cell differentiation, which require Ca^2+^ for their activation [36]. Concept of PKC-mediated chondrogenesis has evolved, starting from the first discoveries of PKC isoform expression and activity. Signaling components upstream and downstream of PKC, leading to the stimulation of chondrogenic differentiation, are also discussed [36]. A study on mechanical compression (0.1 MPa for 1 h) applied in the presence or absence of inhibitors or antagonists of the phosphoinositol and Ca^2+^/calmodulin signaling pathways on explants from bovine articular cartilage has demonstrated the importance of intracellular Ca^2+^ in aggrecan mRNR synthesis [37]. The transduction of the compression-induced aggrecan mRNA-regulating signals was shown to require Ins(1,4,5)P3- and Ca^2+^/calmodulin-dependent signaling processes in bovine articular chondrocytes [37].

Enhancement of chondrogenic differentiation-associated gene expression by intracellular calcium has been demonstrated in hMSCs cultured on 3D scaffolds [38]. Increased expression of the α-1 subunit of VOCCs at both mRNA and protein levels during chondrogenic differentiation have been reported, which is important in enhancement of Ca^2+^ influx for oscillations [31,32], as it forms the channel pore [19,39]. Involvement of calcium influx through the T-type voltage-dependent CaV3.2 calcium channel in chondrogenesis has been demonstrated in a tracheal cartilage model [40]. It was demonstrated, that the lack of CaV3.2 in mice (Cav3.2(−/−)) resulted in an incomplete formation of cartilaginous tracheal support, while the overexpression of this protein resulted in the enhanced chondrogenesis [40]. Nevertheless, the expression and role of VOCCs or TRPV during the process of chondrogenic differentiation of MSC still needs to be elucidated. Firstly, VOCCs and TRPV functions in non-excitable cells are still unclear, especially in the undifferentiated stem cells. Secondly, it seems likely that the expression of VOCCs and TRPV depends not only on the cell type, but also on the state of its differentiation. Finally, VOCCs are in their active conformation only after cell membrane depolarization, which can be caused by a number of external factors, including electric current, magnetic field, and mechanical stimulation, suggesting that these factors might contribute to the chondrogenic efficacy in vitro, see Table 2.

## 7. Stimulation of Chondrogenic Differentiation with Electric Fields

Electrical stimulation (ES) is an effective inductor that guides the development and regeneration of many tissues. It can lead to the secretion of various growth factors by different cells, including TGF-β [44]. ES promotes chondrogenic differentiation and cartilage maturation, however the specific chemical mechanisms that positively control these changes remain elusive [44].

It is well-known, that ES, when applied to a cell suspension, induces a reversible permeabilization of the plasma membrane, leading to the induction of electrically mediated stress in the cells (electropermeabilization, electroporation). This permeabilized state may persist up to several minutes, followed by the self-recovery of the cells. During electropermeabilization, the organization of the cell plasma membrane is affected and transmembrane ion transport is induced, resulting in a burst of intracellular Ca^2+^ [45]. Several studies have demonstrated efficacy of the electric stimulation on bone marrow-derived MSCs, which caused an increase in cytosolic Ca^2+^, leading to activated calmodulin and an increase in TGF-β mRNA expression [35,36]. Furthermore, the ES improved chondrogenic differentiation potential of MSCs by increase in intracellular Ca^2+^ through VOCC (Figure 1).

Chondrogenic differentiation of MSCs has been shown to be efficiently induced using exclusively ES, even in the absence of growth factors [44]. ES enhanced expression levels of classical chondrogenic differentiation markers, including type II collagen, aggrecan, and SOX9, while decreased type I collagen levels. Since ES directly regulates VOCC and can drive Ca^2+^ oscillations in MSC by modulating VOCCs, their implication in chondrogenesis has been suggested [44,45,46]. Application of 1 kHz, 20 mv/cm of ES for 20 min daily (for seven days) led to the chondrogenesis induction in ADSCs, as determined by the increased expression of collagen type II and *SOX9* genes and the partial inhibition of the expression of type I and X collagens [41].

## 8. Magnetic and Electromagnetic Fields Stimulate Chondrogenic Differentiation via Calcium Channels

The increase in intracellular calcium concentration under the application of magnetic field (MF) was reported more than 20 years ago when the effect of an imposed 60 Hz magnetic field on T lymphocytes was investigated [47]. Although it has been a long time since this was reported, little data has been published on stem cell differentiation under static magnetic fields. As living organisms are constantly in motion, a changing MF must always be associated with a changing electric field, according to Faraday’s law, which states that a MF will interact with an electric circuit to produce an electromotive force [48].

The potential relation between static magnetic field, the increased intracellular calcium and improved cell differentiation capacity is still not clear. Although several studies propose that MF might affect cell membrane polarization, resulting in VOCC activation and an increase of intracellular Ca^2+^, these predictions are still under investigation (Figure 1). Magnetic field at 50 Hz, 20 mT inhibited the growth of hBMMSC, while stimulated osteogenic differentiation of these cells [43]. Exposure of hBMMSC cultures to sinusoidal extremely-low frequency electromagnetic fields (EMF) (15 Hz, 5 mT), resulted in high collagen type II expression and GAG content, indicating that EMF has the potential to stimulate and maintain chondrogenesis of hBMMSCs [42]. Although intracellular calcium was not assessed in that study, its involvement in the efficient chondrogenic response seems very likely, as another study has demonstrated stimulatory effects of EMF at 16 Hz on cytosolic calcium levels [49].

Electromagnetic stimulation (EMS) of VOCC gained even more scientific attention as compared to ES, as EMF induced efficient enhancement of osteogenesis and chondrogenesis of MSCs with no negative effects documented so far. Out of twenty three different studies that demonstrated how EMF exposures activate VOCCs, as reviewed in 2013, the majority implicated L-type VOCCs [8]. The possible mechanism of action may be related to nitric oxide levels that are produced through the action of the two Ca^2+^/calmodulin-dependent nitric oxide synthases—nNOS and eNOS. Other studies suggest associations of VOCC activation to the increased cGMP and protein kinase G activity, leading to increased bone growth [8]. Furthermore, exposure to EMF resulted in the increased calcium current inside VOCCs and shorter time being needed for Ca^2+^ to pass the channel [50].

VOCCs are the key signal transducers of electrical excitability, converting the electrical signal of the action potential in the cell surface membrane to an intracellular Ca^2+^ transient [19]. In 2015, Ross with colleagues explained how specific EMF parameters (frequency, intensity, and time of exposure) significantly regulate MSCs chondrogenic differentiation in vitro. The effect of EMF (15 Hz, 5 mT, for 45 min every 8 h) significantly increased the chondrogenic markers glycosaminoglycans (GAGs) and collagen type II in human cell models [42]. Noteworthy, the same 15 Hz frequency stimulates MSCs osteogenesis as well, only under a different strength of the field. Since chondrogenic differentiation of MSCs can undergo hypertrophy, it is not surprising that these two tissues respond to the same frequency, however, for the stimulation of chondrogenic responses, the field must be induced for a longer time [48]. TRP channels (TRPC1 and TRPV4 specifically) have also been shown to become activated due to exposure to pulsed electromagnetic field (PEMF) stimulation. This kind of stimulus induces chondrogenic differentiation by mediating the influx of calcium through the membrane. However, the optimal chondrogenic effect of PEMFs could only be achieved when they are administered in brief low-intensity bursts. To reveal the pathway by which the calcium influx was generated, D. Parate and colleagues investigated whether it was due to the effect on L-type VOCC or TRP channels and found that PEMF-induced chondrogenesis was not affected by the addition of VOCC antagonist nifedipine to the differentiation medium [10]. However, in that study, Nifedipine was used at relatively low dosage, 1 µM. On the other hand, during the repeated PEMF exposure, blocking TRPV4 reversed the inhibition of chondrogenic differentiation while blocking TRPC1 did not, which suggests that TRPV4 plays a more important role in the initiation of chondrogenesis [10].

Therefore, in order to understand the mechanism of action of ES, EMF, or magnetic field on either cell type and mechanotransductive Ca^2+^ channels, and, of course their potential in developing novel therapies based on cell chondrogenic differentiation capability, first of all the parameters, such as frequency, intensity, and time of exposure have to be optimized for either living system where the experimental and environmental conditions are clearly analyzed, thereby allowing for the optimization of a treatment that may have beneficial regenerative effects.

## 9. Stimulation of Chondrogenic Differentiation by Mechanical Forces

Mechanical forces are crucial factors in the maintenance of cartilage in vivo [51]. Chondrocytes are mechanosensitive cells which possess a rich channelome consisting of a large amount of active ion channels. Positive influence of mechanical loading on cartilage ECM component synthesis was repeatedly demonstrated, suggesting its significance in cartilage formation [52,53]. However, while mechanical stimuli can improve growth and properties of cartilage tissue, it also remains one of the major risk factors in the formation of OA [33]. One of the earliest means of chondrocyte response to mechanical-load is an increase in intracellular Ca^2+^ levels [54]. Several types of ion channels, including TRPV4, VOCCs, and others have been recently demonstrated to play critical roles in controlling the intracellular Ca^2+^ responses of chondrocytes in the loaded cartilage [55]. Engineers and scientists are designing protocols to apply mechanical stimulation, including dynamic compression, fluid shear, tissue shear, and hydrostatic pressure seeking to stimulate ECM production in chondrogenesis and cartilage models [56]. The use of more advanced bioreactors, such that incorporate shear and other components of loading, enhances the chondrogenic response of hMSCs to mechanical loading and better mimics cartilage in vivo environment [51]. Moreover, calcium signaling in bone marrow MSCs in response to hydrostatic pressure, including increased chondrogenic gene expression, chondrocyte matrix production, and the suppression of hypertrophy markers, was associated to VOCCs activity [38]. Inhibition of VOCCs with 10 μM verapamil in that study, resulted in a reduction of chondrogenic response in MSCs to hydrostatic pressure. This suggests that appropriately applied mechanical stimulation positively influences MSC-induced chondrogenic differentiation, ECM deposition, and the mechanical properties of the forming in vitro cartilage. Furthermore, mechanotransduction involves conversion of mechanical load into biochemical responses with possible assistance of VOCCs and other mechanosensitive Ca^2+^ channels, resulting in further Ca^2+^ influx, see Figure 1. In addition to VOCCs, TRP channel activation is also mediated by receptors and ligands or stimulated directly through changes in temperature, phosphorylation, and mechanical stimuli [24]. Mechanosensitivity seems to be one of the aspects that is shared by most TRP channels and that some of the chemical factors that lead to TRP channel activation may work by affecting membrane curvature, either directly or via a cascade of reactions, rather than affecting the channels directly. TRPC1 specifically was shown to react to membrane stretch induced by hypo-osmolarity [57]. A notable example of TRP channel mechanosensitivity is the fact that TRPV1, TRPV2, and TRPV4 have all been shown to cause increased Ca^2+^ influx in response to mechanical stimuli in mice models [58]. Taken together, different mechanical loading actually enhances the chondrogenic differentiation of hMSC, however, the channels influencing Ca^2+^ entry and regulating downstream molecular signals to enhance chondrogenesis remain to be clarified.

## 10. Conclusions

### Different Stimuli Result in Increased Intracellular Calcium Leading To Similar Effects

Effective stimulation of chondrogenic differentiation in human mesenchymal stem cells is highly desirable and a clinically relevant process due to the poor regenerative capabilities of cartilage. Current techniques that are proposed to enhance chondrogenic differentiation in vitro often include the use of a range of growth factors—TGF-β, Activin A, and BMP-2. Additional application of different physical stimuli—mechanical, electrical, electromagnetic, or magnetic field might further lead to the enhanced calcium concentration and ultimately to the enhanced activation of chondrogenic gene expression and the formation of extracellular matrix in cartilage. Different studies propose electrical stimulation as the most potential stimuli to activate VOCCs, while others claim that mechanical stimuli is necessary when it comes to cartilage tissue formation in vitro. Magnetic field is still under research, though several studies suggest it as a potential mean in the chondrogenic differentiation capacity of MSCs. Although none of these studies clarify the interplay between the channels in chondrogenic differentiation, in most of the cases, it was associated with the increased calcium influx. The parameters of the applied physical stimulation, including strength, duration, intervals, etc., seem to have the essential impact on their efficacy in stimulation of the ECM production [10]. In conclusion, although this field of research is still in its infancy, the application of physical stimuli may reveal new methods to stimulate chondrogenesis of hMSCs, which in turn, might lead to the development of the efficient therapeutic strategies for OA.

## Figures and Tables

**Table 1:** Classification of voltage-operated calcium channels (VOCCs) based on their structure and activation.

VOCCs	Type:	α1 Subunits:	References:
LVA	T-type	CaV3.1, CaV3.2, CaV3.3	[18,19]
HVA	L-type	CaV1.1, CaV1.2, CaV1.3, CaV1.4	[20,9]
	N-type	CaV2.1	
	P/Q-type	CaV2.2	
	R-type	CaV2.3	

**Table 2:** The effect of external stimuli on chondrogenic differentiation of mesenchymal stem cells.

Stimuli	Cell Type	Application	Effect	Channel/Inhibitor	Chondrogenic Response	References
Electric stimulation	ADSC	Electric stimulation, 1 KHz, 20 mv/cm, 20 min/day, 7 days	Increased expression of chondrogenic differentiation markers	VOCCs	positive	[41]
Electromagnetic stimulation	BMMSC	EMF, 15 Hz, 5 mT, 45 min every 8 h	Increased expression of chondrogenic differentiation markers	VOCCs	positive	[42]
	BMMSC	PEMF, 15 Hz, 2 mT, 10 min, daily	Increased expression of chondrogenic differentiation markers	TRPs/10 μM Ruthenium Red; 100 µM 2-APB	positive	[10]
	BMMSC	EMF, 15 Hz, 5 mT	Increased expression of chondrogenic differentiation markers	Unclear	positive	[42]
Magnetic field	BMMSC	MF, 50 Hz, 20 mT	Inhibited MSC growth	Unclear	negative	[43]
Mechanical forces	Bone marrow MSCs	Hydrostatic pressure, 10 MPa at a frequency of 1 Hz, 4 h/d, 5 d/week for 3 weeks	Enhanced chondrogenic gene expression	VOCCs/10 μM verapamil	positive	[34]

## Abbreviations

BK	Large Conductance Calcium-activated potassium channels
SK	Small Conductance Calcium-activated potassium channels
ECM	Extracellular matrix
ES	Electrical stimulation
ADSC	Adipose-derived stem cells
EMS	Electromagnetic stimulation
EMF	Electromagnetic field
GAG	Glycosaminoglycan
PEMF	Pulsed electromagnetic field
MF	Magnetic field
Ca^2+^	Calcium ions
CaM	Calmodulin
HVA	High-voltage activated
InsP3R	Inositol 1,4,5-triphosphate receptor
KV	Voltage activated potassium channels
KATP	ATP Sensitive Potassium channels
LVA	Low-voltage activated
MSCs	Mesenchymal stem cells
Na^+^	Sodium ions
OA	Osteoarthritis
P2X4	Purinergic receptors, associated with mechanotransduction
PIP2	Phosphatidylinositol 4,5-bisphosphate
PKC	Protein kinase C
RyR	Ryanodine receptor
TMs	Transmembrane domains
TRP	Transient receptor potential channels
VOCCs	Voltage-operated calcium channels
